# Virginia Dental Society—Annual Meeting

**Published:** 1867-12

**Authors:** W. Leigh Burton

**Affiliations:** Secretary.


### *Virginia Dental Society—Annual Meeting.*

The annual meeting of the Virginia Dental Society was held on the 21st of October at the office of Dr. John Mahoney.

The old officers were reelected, with the exception of Dr. John G. Wayt, who declined being a candidate for the presidency. The following dentists are officers of the Society:

Dr. R. N. Hudson, president; Dr. G. W. Jones, vice-president; Dr. W. Leigh Burton, secretary; and Dr. J. Edward Chase, treasurer. Executive Committee: Drs. J. Hall Moore, Joseph Woodward, and George B. Steel.

Besides other business before the Society, the propriety of abolishing the present tariff of charges for professional services having been discussed, on motion it was

*Resolved*, that the tariff of charges adopted at a meeting of this Society March 2, 1867, be, and the same is hereby, abolished.

W. Leigh Burton,
*Secretary.*

